# Ultrastructure and complex polar architecture of the human pathogen *Campylobacter jejuni*

**DOI:** 10.1002/mbo3.200

**Published:** 2014-07-25

**Authors:** Axel Müller, Morgan Beeby, Alasdair W McDowall, Janet Chow, Grant J Jensen, William M Clemons

**Affiliations:** 1 Division of Chemistry and Chemical Engineering, California Institute of Technology1200 E. California Blvd., Pasadena, California, 91125; 2 Division of Biology, California Institute of Technology1200 E. California Blvd., Pasadena, California, 91125; 3 Beckman Institute, California Institute of Technology1200 E. California Blvd., Pasadena, California, 91125; 4 Howard Hughes Medical Institute, California Institute of Technology1200 E. California Blvd., Pasadena, California, 91125

**Keywords:** Acidocalcisomes, *Campylobacterales*, chemoreceptors, cryoelectron tomography, cryo-EM, food poisoning, polyphosphate storage granules

## Abstract

*Campylobacter jejuni* is one of the most successful food-borne human pathogens. Here we use electron cryotomography to explore the ultrastructure of *C. jejuni* cells in logarithmically growing cultures. This provides the first look at this pathogen in a near-native state at macromolecular resolution (∼5 nm). We find a surprisingly complex polar architecture that includes ribosome exclusion zones, polyphosphate storage granules, extensive collar-shaped chemoreceptor arrays, and elaborate flagellar motors.

## Introduction

*Campylobacter* spp. are the major bacterial cause of food poisoning in the United States with ∼45,000 annual cases identified and an estimated 800,000 total infections (Crim 2013). This results in an estimated annual cost of 1.7 billion dollars in the United States alone (Batz et al. 2012). *Campylobacter jejuni*, responsible for 90% of *Campylobacter* spp. infections (Frost et al. 1999), lives commensally in the guts of birds contaminating most raw poultry products, the largest route of infection (Vugia et al. 2006; Mullner et al. 2009). Illness manifests as fever, cramps, abdominal pain, and bloody diarrhea. One in 1000 campylobacteriosis cases results in Guillain–Barré syndrome (GBS) (Tam et al. 2007; Ternhag et al. 2008), an autoimmune disease that causes severe damage to the peripheral nervous system leading to potentially life-threatening paralysis.

Despite its medical importance, little work has been done to characterize the ultrastructure of *C. jejuni*. The first visualization of *C. jejuni*, then known as *Vibrio fetus*, was by light microscopy, where the shape is described as fine wavy or sinuous lines of various lengths (Smith 1919). Significantly later, electron micrographs were produced using chemical fixation, dehydration, and metal staining resulting in high-contrast images (White 1967; Buck et al. 1983), but also well-known artifacts. In these studies, *C. jejuni* were seen with both helical rod and coccoid shapes. Each rod-like cell was amphitrichous, one flagellum at each pole, with the flagella emerging from concave depressions in the cell wall (Pead 1979).

In this report, we use electron cryotomography (ECT) to clarify the ultrastructure of *C. jejuni* originating from a patient suffering from GBS. In ECT, specimens are flash frozen leaving them in a fully hydrated, near-native state. This approach revealed a complex polar architecture including a large flagellar motor, a conical chemoreceptor array, polyphosphate storage granules, and surprising polar ribosome exclusion zone (REZ).

## Experimental Procedures

### Growth conditions

Freeze dried *C. jejuni* ssp. *jejuni* ATCC® 29428™ was reconstituted according to manufacturer's instruction and plated on Brucella agar supplemented with 5% sheep blood and grown at 37°C under microaerophilic conditions (oxygen concentration of 1.5% and concentrations of hydrogen and carbon dioxide of 10%). For long-term storage, cells were flash frozen in 25% glycerol. For growth in liquid culture, *C. jejuni* from glycerol stocks was streaked onto Brucella agar supplemented with 5% sheep blood and incubated under microaerophilic conditions. After 48 h, one colony was transferred from the plate into a 25 mL tissue culture flask containing 5 mL brain–heart infusion (BHI) medium and grown to log phase (OD_600_ of 0.5) at 37°C under microaerophilic conditions for 20 h. The viability of the cells was confirmed by light microscopy where samples were viewed under 1000-fold amplification.

### Sample preparation

Sample preparation closely followed established procedures (Iancu et al. 2007). In brief, 4 *μ*L of a 10 nm colloidal gold (Sigma Aldrich, St. Louis, MO, USA) in 5% Bovine serum albumin (BSA) was added to 16 *μ*L of a *C. jejuni* culture that had been allowed to grow to an optical density of 0.5. Three microliters of this mix were then placed onto a glow discharged carbon-coated R 2/2 Quantifoil grid in a Vitrobot (FEI Company, Hillsboro, OR). Prior to this a 10 nm colloidal gold suspension in 5% BSA solution was added to the Quantifoil grid and allowed to dry. The temperature in the Vitrobot™ chamber was kept at 22°C with 100% humidity. Placing the sample onto the grid was followed by a 1 sec blot with an offset of −1.5°, a drain time of 1 sec, and plunge frozen in a mixture of liquid ethane (63%) and propane (37%). The frozen grids were than stored in liquid nitrogen until further use.

### Data collection and 3D reconstruction

Single-axis tilt series from −60° to 60° with images were collected in 1° increments and an underfocus of 12 *μ*m using a 300 keV FEI Polara FEG TEM controlled by Leginon software (Suloway et al. 2009) and the cumulative dose was not allowed to exceed 200 e Å^2^. The images were recorded on a 4096 × 4096 pixel Ultracam (Gatan, Pleasanton, CA) at a magnification of 22,500 (0.98 nm/pixel). The IMOD software package (Kremer et al. 1996) was used to calculate 3D reconstructions.

### Statistics

From electron tomograms recorded at a magnification of 3000×, 100 *C. jejuni* cells were randomly selected. The images had a resolution of 2048 × 2048 pixels. Measurements were carried out in the raster graphics editor GIMP. For measurements of cell length the path connecting the poles along the equator was chosen, widths were measured on five different locations along the cell, and averaged. R (R Foundation for Statistical Computing 2008) was used for statistical analysis and for plotting graphs.

## Results and Discussion

### Overall shape

For this study, a strain of *C. jejuni* was chosen for its medical relevance, as it was originally isolated from the feces of a human infant diagnosed with GBS. Cells were grown to logarithmic phase in BHI medium under microaerophilic conditions then first observed under a light microscope with 1000-fold magnification as rod shaped and motile. They swam rapidly in straight lines through the view field of the microscope without tumbling or changes in direction.

**Figure 1:**
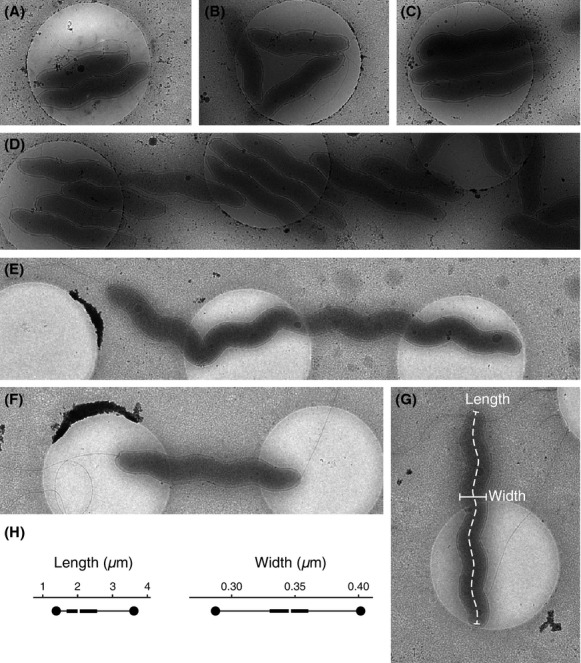
Low magnification cTEM images of *Campylobacter jejuni* on carbon grids. For scale, the diameter of the holes is 2 *μ*m. (A–G) A survey of *C. jejuni* cells. At this magnification overall shape and some intracellular features are visible. (E) shows a particularly long cell where a hole on the grid likely forces a bend. (G) The lines define the length and width measurements. (H) Bar charts illustrating the maxima and minima (circles), first and third quartiles (beginning and end of rectangle), and medians (gap) of length and width measured in 100 *C. jejuni*. To determine the average width five measurements were performed in each cell.

Separately, the logarithmic cells were prepared for electron microscopy by the addition of colloidal gold fiducial markers and then flash frozen on carbon grids containing 2 *μ*m holes. Grids were first viewed using transmission electron cryomicroscopy (cTEM) at 3000× magnification, as cell shapes are best characterized at this level (Fig.1A–G). Cells vary in length with many spanning across multiple holes in the grid. They are slender with a gentle, spirillum shape and in the typical bacteria the flagella are clear at both poles with granules strongly visible. Flagella are extremely long and cannot be fully traced, as many flagella overlay in the frame of view. Some parts of cells on the carbon show signs of slight deformations consistent with the bacterium having some flexibility; in fact, a few cells show bends likely caused by lying across holes on the grid (Fig.1E).

A noticeable feature of these preparations was that many of the bacteria were organized into groups on the grid (Fig.1A–D). Typically, these were tight associations along the length of the cell in a side-by-side fashion. This required a tight fit with the cells matching their helical turns suggesting that the periodicity of the helical turn is tightly controlled. Upon closer inspection, tomograms for these groupings (see below) do not reveal any direct interactions between cells; however, the width of the periplasmic space appears more irregular when two cells are in proximity. This behavior appears to be a characteristic of *Campylobacter*, as it has not been observed in previous studies from a sizable and diverse set of other bacteria (Chen, 2011).

Using the cTEM images, a statistical analysis of 100 *C. jejuni* was performed. Typical *C. jejuni* have a single flagellum and flagellar motor at each pole. *Campylobacter jejuni* with a single flagella are likely to have undergone recent cell division. The length, defined as the distance along a path through the middle of a bacterium (Fig.1G), ranges between 1.4 and 7.01 *μ*m with a median of 2.00 *μ*m, first and third quartiles of 1.72 and 2.44 *μ*m, respectively (Fig.1H). It is likely that longer cells are in the process of cell division. Width was measured as the average of five independent measurements per cell of the diameter of the outer membrane (Fig.1G) and varies between 0.255 and 0.409 *μ*m with a median of 0.346 *μ*m, first and third quartiles 0.331 and 0.360 *μ*m, respectively (Fig.1H).

### General architecture

To explore the finer ultrastructure of *C. jejuni*, full 3D cryotomograms at higher magnification were recorded. Tilt series covering 120° of perspective were acquired of select cells within the same samples used for cTEM. Predominantly, cells with their polar region fully within the holes of the grid were chosen. Consequently, whole cells examined tended to be shorter; however, in comparison to polar features of longer cells there were no obvious differences.

**Figure 2:**
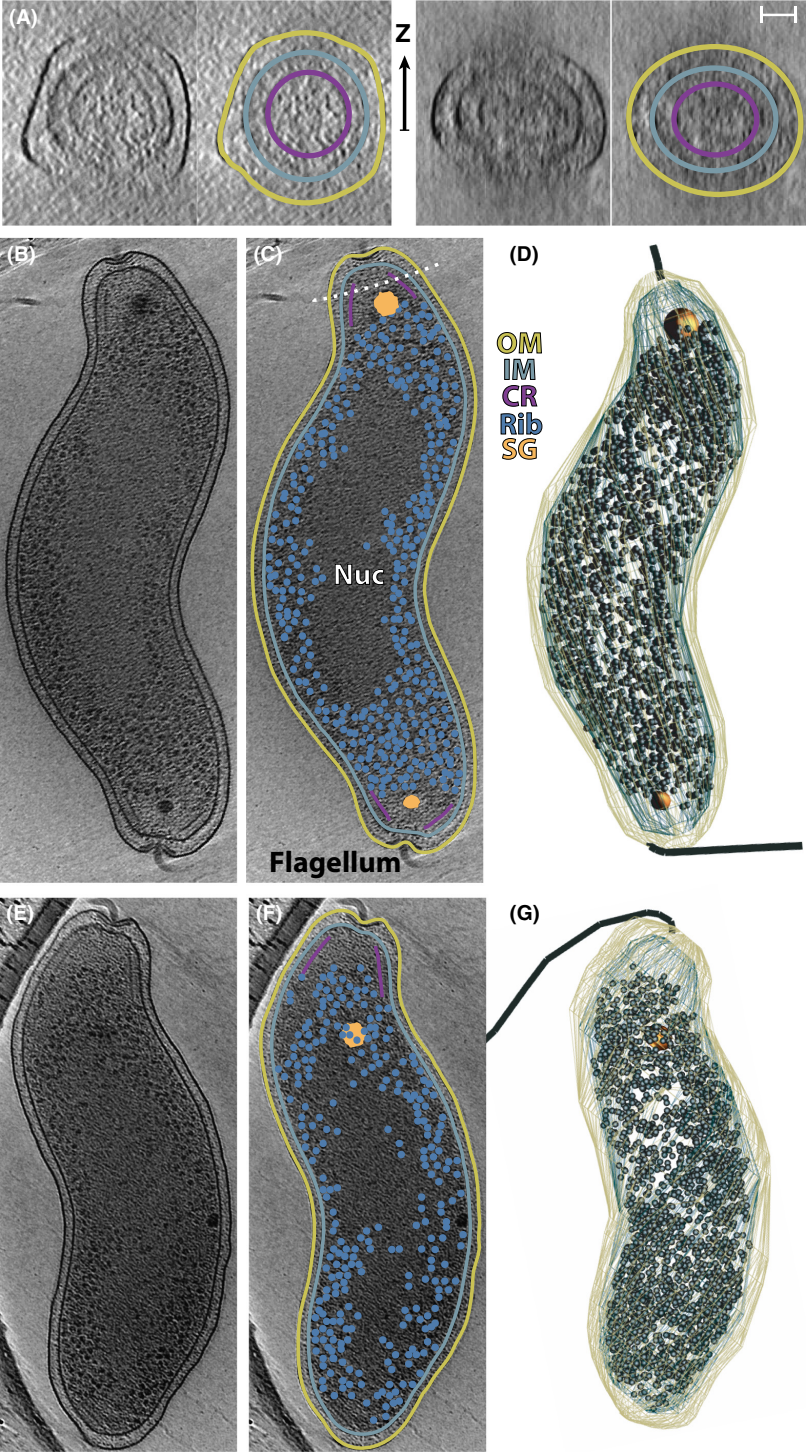
Ultrastructure of *Campylobacter jejuni* by ECT. In A–C and E and F, duplicate images are highlighted on the right: outer membrane (OM, gold), inner membrane (IM, light blue), chemoreceptors (CR, purple), ribosomes (Rib, blue spheres), storage granules (SG, orange), and nucleoid (Nuc). The scale bar in A is 100 nm (also corresponds to [B–G]). (A) Two tomographic slices through a *C. jejuni* cell oriented to highlight how the chemoreceptor arrays form a complete collar (see dotted line in [B] for approximate location). The cells, due to their slender nature, experience only minor distortion during sample preparation. The arrows indicate the direction of the electron beam along which resolution is attenuated. (B) Tomographic slice of a *C. jejuni* cell. (C) A cartoon highlighting the various features of (B). Note the central and polar ribosome exclusion zones (REZ). In this figure, the variability in the thickness of the periplasm can also be seen. (D) 3D model of *C. jejuni* based on the full tomograms from (B). The two membranes, flagella, storage granules, and ribosomes are highlighted. Ribosomes were manually identified in the tomogram. The left model has two fully developed poles, with flagella and polar REZs. (E–G) Similar to (B–D) for a *C. jejuni* cell with a single flagellum on one pole that maintains the common polar features. The opposing pole is missing the typical features, perhaps the result of a recent cell division.

Because of surface tension in the thin fluid layer in which the cells are suspended on the grid, flexible cells can become flattened (Tocheva et al. 2010). For *C. jejuni*, the cross-sections exhibit an almost circular shape (Fig2A), perhaps because of the relatively small width or robust structure, justifying the width measurements reported above. The cross-sections also illustrate how the well-understood “missing wedge” of tilt limitations attenuate resolution along the *z*-axis, obscuring thin features such as membranes when they lie in the *xy* plane (Fig.2A) (Gan and Jensen 2012).

A few smaller cells were caught entirely suspended within a hole in the carbon grid (Figs.1A, 2B). This enabled the description of the key morphological features from *C. jejuni* in the context of the entire cell. Using the tomogram of Figure 2B, a three-dimensional model was created based on the features seen in individual slices. A second cell is also modeled that lacks polar features at one end, likely because of a recent cell division (Fig.2C).

**Figure 3:**
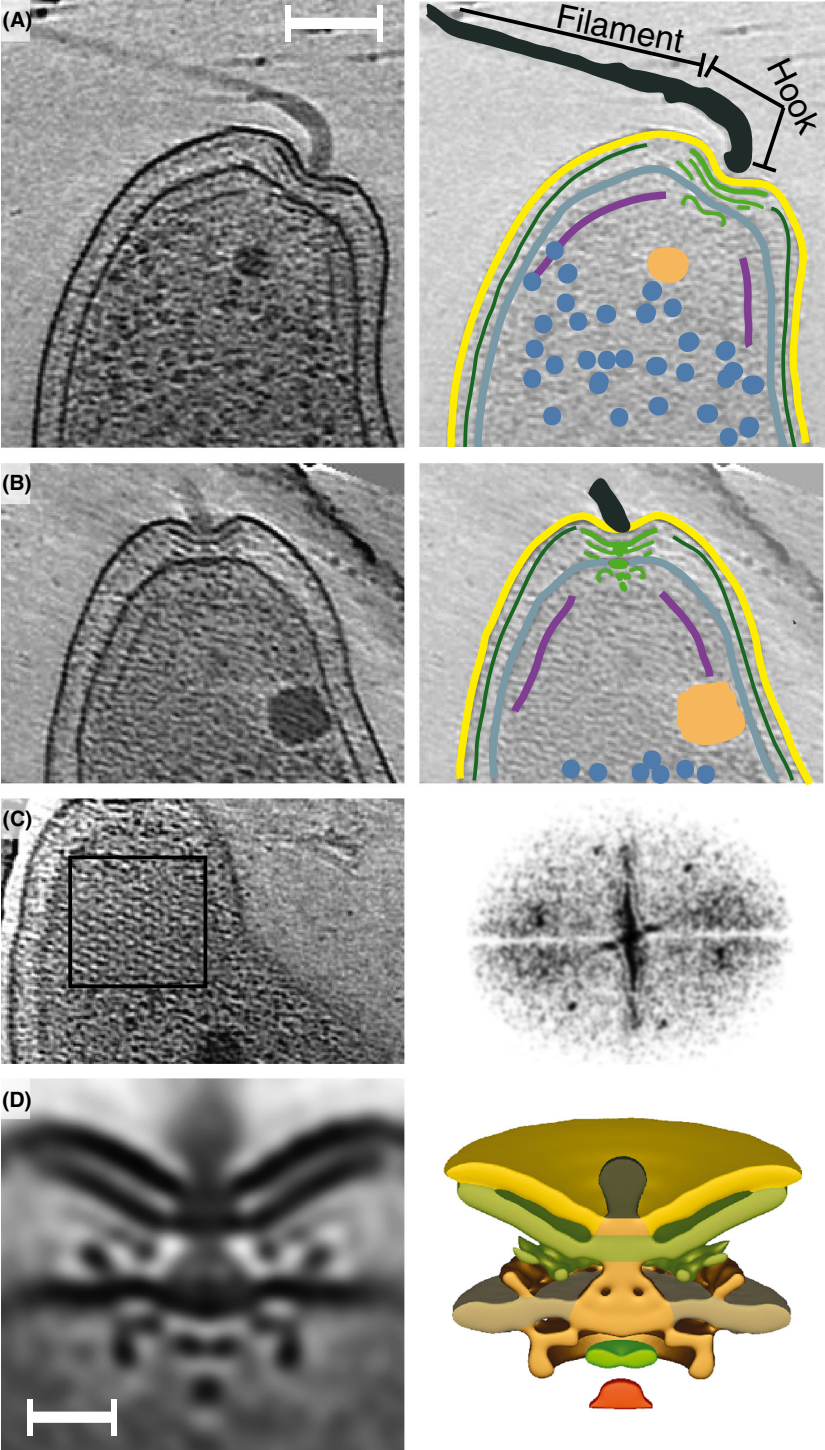
Polar architecture. (A, B) Tomographic slices and cartoons of two *Campylobacter jejuni* poles. Colors and labels are as above, with the addition of the flagellar motor (green) and flagellar hook and filament labeled. The scale bar in A is 100 nm and applies to (A–C). (C) (left) Tomographic slice providing a top view of a chemoreceptor array. The black box shows the region used to calculate the Fourier transform (right) demonstrating the hexagonal lattice. (D) (left) Slice through a subtomogram average of the *C. jejuni* flagellar motor showing its unusual periplasmic structure. (right) Isosurface of same subtomogram average. Membranes (same color code as in Figure2), FliI (red), FlhAC (green), and as yet unidentified components (orange), scale bar is 25 nm.

The periplasm, bisected by the peptidoglycan, is characterized by a low contrast relative to the cytoplasm due to the lower density of macromolecules (Fig.3B). At the poles, the periplasm has a dramatically irregular width due to the presence of the flagellar motor (Fig.3). Elsewhere the width of the periplasm is less variable (Fig.2B), although the outer membrane exhibits wrinkles not seen in the smooth inner membrane. Periplasmic width variation has been noted before; in cTEM of *Escherichia coli* and *Pseudomonas aeroginosa* the periplasmic width varies up to 10% (Matias et al. 2003), although some of the variation is attributable to distortions caused by sample preparation.

### Polar features

The two polar flagella provide the motility that allows *C. jejuni* to swim in the directed fashion seen by light microscopy. Gene deletions that disrupt the flagella are unable to colonize in host models demonstrating the importance of motility in pathogenicity (Nachamkin et al. 1998; Guerry 2007). The flagella are composed of a hook, junction, filament, and cap as previously noted (Galkin et al. 2008; Hendrixson 2008), consistent with the features seen in the tomograms (Fig.3A). Viewed in the wider field of cTEM, the flagella are typically much longer than the body of the bacterium (Fig.1F and G) with the filament measuring ∼24 nm in thickness. Only a minority of the cells observed have a single flagellum suggesting that the flagellum and motor assemble quickly after cell division. The presence of locomotion machinery at each pole leads to open questions about how they work synergistically. Tumbling and swimming are well described in organisms such as *E. coli*; however, this model needs modification to be applicable to amphitrichous bacteria. During the limited observations made here by light microscopy, none of the *C. jejuni* tumbled during the time that they were within the field of vision. Based on current literature, it remains unclear how bipolar flagella coordinate to produce directed motility.

One of the features distinguishing *C. jejuni* from other species that have been visualized by ECT is that the flagella emerge from crater-like structures (best seen in Fig.3). This unusual polar structure was first noted in negative stain where it was simply described as a “concave depression” (Pead 1979). In the tomograms, it is clear that the structure is the result of a thickening of the periplasm to accommodate the large flagellar motor (Fig.3A and B). A protein ring on the periplasmic side of the outer membrane likely contributes to this (Chen et al. 2011).

Rod-shaped bacteria with flagella typically assemble chemoreceptor arrays near their poles. In *C. jejuni* the arrays are large structures that enclose the pole just inside the inner membrane (Fig.2B). Cross-sections through the full cone of chemoreceptors appear as pronounced lines in tomographic slices (Figs.2A and B, 3A and B). Fourier transforms clearly demonstrate the expected 12-nm hexagonal lattice (Fig.3C) (Briegel, 2009). As reported previously, the cones start near the edge of the flagellar motor and extend ∼200 nm along the length of the cell resulting in a surface area of ∼75 nm^2^ per array corresponding to roughly 7500 receptors (Briegel et al. 2009). How these pairs of large receptor arrays communicate during chemotaxis is unknown. As in other organisms, we presume they communicate to the flagellar motors via the diffusing signaling protein CheY. The large, conical chemoreceptor array immediately adjacent to and surrounding the motor may then provide a focused and rapid signal. The small size of *C. jejuni* makes it likely that the two arrays (at opposing poles) experience the same concentrations of chemotactic molecules; however, it remains to be determined how these responses would be coordinated. Of note, the polar region enclosed by the chemoreceptor array excludes large molecules (see below), and may affect signal diffusion.

Bacterial cells are filled with ribosomes. In *E. coli* there are ∼27,000 ribosomes/*μ*m^3^ (Bakshi et al. 2012). This is consistent with the density we estimate for *C. jejuni* based on a manual count of ribosomes (∼25,000 ribosomes/*μ*m^3^) (Fig.2). In *C. jejuni*, they are typically found throughout the entire cytoplasm except for the nucleoid region where they are absent, creating a REZ (Fig.2B and C). Surprisingly, a second REZ was also noted at the poles within the chemoreceptor array cones (Figs.2B and C, 3A and B). This polar REZ is reminiscent of structures seen previously for an overexpression mutant of PopZ, a protein thought to anchor chromosomal origins to the pole, in *Caulobacter crescentus* (Ebersbach et al. 2008). Presumably a dense aggregate of PopZ prevents diffusion of ribosomes into this area. The *C. jejuni* polar REZ does not require an additional membrane nor does its genome encode for a PopZ homologue. The comparison to the PopZ mutant suggests that diffusion of large molecules into the polar REZ is prevented by a protein meshwork or aggregate, although other complex structures, such as glycans, cannot be ruled out. The role and nature of this novel REZ are major questions that await elucidation.

Most of the examined *C. jejuni*, all from exponentially growing cultures, had one or two granules with diameters between 25 and 70 nm often at the periphery of the polar REZ (Figs.1, 2B and C, 3A and B). While this positioning likely has functional implications, its physical cause may simply be exclusion from the polar REZ and nucleoid (Beeby et al. 2012). Based on appearance (compared with previous studies), the presence of polyphosphate synthesis genes and the absence of genes encoding microcompartments (Jorda et al. 2013), we suggest that these granules are polyphosphate storage granules (also known as acidocalcisomes) (Comolli et al. 2006; Beeby et al. 2012; Tocheva et al. 2013). Polyphosphate storage granules are electron dense (visible as darker particles in tomograms) due to the high concentrations of phosphate complexed by counter ions such as magnesium and calcium. Organisms utilize polyphosphate to regenerate nucleotide triphosphates (Kuroda et al. 1997); therefore, polyphosphate storage granules are regarded as energy storage granules. In eukaryotes, polyphosphate storage granules are found in membrane bound organelles and a similar architecture has been suggested in bacteria (Docampo et al. 2005). Other types of membrane-encapsulated particles have been clearly visualized by ECT (Komeili 2006); however, in *C. jejuni* the polyphosphate storage granules are not enclosed by membranes. The presence of polyphosphate storage granules close to the polar REZ boundary may suggest an additional involvement in motility. In several human pathogens, polyphosphate synthesis genes are linked to virulence and are thought to play a role in stress responses and survival during stationary phase (Rao and Kornberg 1996). For these organisms, deletions of *ppk* genes, required for polyphosphate generation and utilization, results in bacteria with no apparent phenotypes in rich liquid media; however, there is a significant loss in motility on solid media (Rashid and Kornberg 2000). In *C. jejuni*, knockout mutants of *ppk*1 and *ppk*2 are similar, with impaired pathogenesis and stress responses, particularly in cell invasion, a process presumably dependent on motility (Candon et al. 2007; Gangaiah et al. 2009). This suggests a possible link to the polar location.

A detailed understanding of the ultrastructure and life cycle of *C. jejuni* will be useful in the search for new treatment strategies to combat this pathogen. This report presents a critical missing component, providing new insights into the complex chemoreceptor arrays, the presence of polyphosphate storage granules, and the surprising polar REZ. Each of these components likely plays a crucial role in *C. jejuni* infections. The large chemoreceptor arrays that form a cap at the poles may allow the organism to sense environmental changes quickly. The putative protein aggregate that forms the polar REZ may contribute to swift signal transduction by keeping the cytoplasmic molecules involved in this process localized to the poles. The unusually large motor and flagellum allow *C. jejuni* to penetrate the viscous mucous layer and ultimately invade host intestinal epithelia. Polyphosphate storage granules likely provide energy storage needed to transition between environments such as the nutrient-poor latent stage experienced between hosts. Further understanding of the features described here may open the road to novel methods to keep this pathogen out of our food chain.
